# Comparison of three primer pairs for molecular sex determination in Eurasian pygmy owls (*Glaucidium passerinum*)

**DOI:** 10.1038/s41598-024-65157-3

**Published:** 2024-07-16

**Authors:** Simona Stehlíková Sovadinová, Chahrazed Mekadim, Erkki Korpimäki, Jakub Mrázek, Marek Kouba

**Affiliations:** 1 Department of Ethology and Companion Animal Science, Faculty of Agrobiology, Food and Natural Resources, Czech University of Life Sciences Prague, Kamýcká 129, Praha 6 – Suchdol, 165 00 Czechia; 2 Laboratory of Anaerobic Microbiology, Institute of Animal Physiology and Genetics, Czech Academy of Sciences, Vídeňská 1083, Praha 4 – Krč, 142 20 Czechia; 3 Section of Ecology, Department of Biology, University of Turku, Turku, 20014 Finland; 4 Department of Game Management and Wildlife Biology, Faculty of Forestry and Wood Sciences, Czech University of Life Sciences Prague, Kámycká 129, Praha 6 – Suchdol, 165 00 Czechia

**Keywords:** 2550F/2718R, Birds of prey, CHD1F/CHD1R, Laboratory sexing, P2/P8, Sequencing, Biological techniques, Genetics, Zoology

## Abstract

Bird sex determination is fundamental in various ecological and biological studies, although many avian species cannot be sexed visually due to their monomorphic and/or monochromatic appearance. Thus, reliable laboratory methods for sexing are a prerequisite. Most avian nestlings lack sex-related signs, including the Eurasian pygmy owl (*Glaucidium passerinum*). We performed laboratory sex determination analysis of this species using blood samples of 242 juveniles and nine adults. It relied on the qPCR of the specific intron from the chromo-helicase DNA-binding protein 1 gene. We tested three primer sets, the P2/P8, 2550F/2718R, and CHD1F/CHD1R, commonly used for bird laboratory sexing. The outcomes were displayed on an agarose gel electrophoresis and a plot from melt curve analysis, which had not been previously conducted in Eurasian pygmy owls. We found that only primer set CHD1F/CHD1R proved reliable, as the only one determined sex with one and two band/s and peak/s on the electrophoresis and the melt curve plot for males and females, respectively. The other two primer pairs failed and depicted one band/peak in all specimens regardless of their sex. Therefore, we recommend performing Eurasian pygmy owls’ laboratory sexing by qPCR with CHD1F/CHD1R primers only.

## Introduction

Methods of sex determination based on morphological features are unreliable in monomorphic and monochromatic birds and virtually impossible for juvenile ones^1–3^. This difficulty arises from the minimal sexual dimorphism in many avian species. In birds of prey (owls and diurnal raptors), adult females are usually larger than males and can be sexed during the breeding season because only females incubate and have a brood patch^4,5^. Newly-hatched and half-grown nestlings of birds of prey do not usually show any sex-related physical characteristics and usually cannot be properly sexed based on body size measurements only^6,7^. However, identifying the sex of newly-hatched chicks holds significant importance in ecological and evolutionary studies, including, for instance, parental sex allocation^8,9^, sex-specific immune responses^10^, sex ratio evolution and mating systems^11–13^, research on the evolution of sexual size dimorphism^14–16^, as well as breeding and conservation management^17–20^.

Nowadays, molecular methods for sex identification are commonly used. The principle of laboratory procedures for bird sexing often hinges on variations in the intron lengths of the conserved chromo-helicase DNA-binding protein 1 (CHD1) gene between the sex chromosomes Z and W. However, methods employing only Z-specific genes have also been developed^21^. These patterns are applicable to most avian species^21–24^. Different protocols are applied to the ratites (e.g., ostriches—Struthioniformes), in which the Z and W chromosomes are essentially homomorphic, i.e., identical in size^21,25,26^. Primer sets specific to the CHD1 gene have been independently developed, with the ones most frequently utilised being P2/P8^27^, 2550F/2718R^28^, and CHD1F/CHD1R^29^. In addition, advanced techniques employ quantitative polymerase chain reaction (qPCR) combined with melt curve analysis and thus offer gel-free, rapid, and high-throughput solutions^30,31^.

Various laboratory sexing analyses have been performed in studies on owl species, such as snowy owl (*Bubo scandiacus*)^32^, Indian scops owl (*Otus bakkamoena*)^33^, Tengmalm’s owl (*Aegolius funereus*)^34^, Southeast Asian barn owl (*Tyto alba javanica*)^3,35^, and Eurasian eagle owl (*Bubo bubo*)^36^. However, before the first molecular sexing of a given species, a primary calibration of the laboratory methods should always be done, and an appropriate primer set should be selected that gives the correct results for the studied species^37,38^.

Despite the many studies on laboratory sex identification of owls, as far as we know, only one has addressed laboratory-based sexing that included the Eurasian pygmy owl (*Glaucidium passerinum*, hereafter PO) among target species^39^. Investigation directly testing multiple primer sets in PO or using melt curve analysis that can offer dependable digital conversion of data, even with minimal DNA concentration, has not been published yet.

PO is a widely distributed species inhabiting semi-open coniferous and mixed forests from Central and Northern Europe east through Siberia to Sakhalin and Northern China^6^. POs lack noticeable external morphological distinctions between males and females, except for a minor reversed sexual size dimorphism with females being larger than males: wing length of breeding females with brood patch averages 106 mm (5th–95th percentiles 103–110 mm), that of males 99 mm (5th–95th percentiles 96–102 mm), tail length of females mean 65 mm (5th–95th percentiles 61–69 mm), males mean 60 mm (5th–95th percentiles 55–65 mm), body mass females represent 77 g (5th–95th percentiles 69–87 g), males represent 60 g (5th–95th percentiles 55–65 g)^40^. With these body measurements, PO ranks among the smallest owls, making their sex determination challenging without specialised techniques^5,6^.

Therefore, this study primarily aimed to assess the effectiveness and reliability of commonly used methods for molecular sex determination in the case of POs. This objective involved validating the suitability of three widely used primer sets, P2/P8, 2550F/2718R, and CHD1F/CHD1R. Additionally, the study aimed to provide a dependable laboratory method based on melt curve analysis, which was not previously conducted for PO sex identification. The final study goal was to sequence all DNA fragments amplified by the primers mentioned above in POs and deliver them to the National Centre for Biotechnology Information database (hereafter NCBI).

## Materials and methods

We worked, in total, with 251 blood samples of POs from 47 broods, of which 242 blood samples were collected from nestlings (juveniles) from 2007 to 2009 and nine samples of adults (seven males and two females) taken in 2009 and 2012. All blood samples were collected in the Kauhava study area of west-central Finland (63° N, 23° E; 50–110 m a. s. l.) during regular nest visits carried out after their finding in local nest-boxes. The study area covered 1000 km^2^ and included 230 coniferous forest patches with two nest-boxes 80–100 m apart suitable for breeding POs^41^.

The sex of adults was initially determined by observing secondary sexual traits and behaviour, providing a basis for verifying laboratory procedures. Adult males were attracted to the vicinity of their nest by playback of whistling con-specific and captured with a telescopic fishing pole with a loop at the top or with mist-net^40,41^. Only parent females have brood patches and incubate and brood the chicks^5,41^. Adult females and nestlings were captured and sampled during their stay in the nest-box. Fifty μl of blood samples from each individual were collected by brachial vein puncture under the wing using sterile needles and capillaries. In the case of nestlings, blood samples were collected ca. 14 days after hatching.

Owls were trapped, handled and ringed under the ringing licence of the Finish Museum of Natural History (Permit No. 524 to EK), as well as blood-sampled under Permit No. 1524/05 approved by the Animal Experiment Committee of the University of Turku and under the approval of the Centre for Economic Development, Transport and the Environment of South Finland (Permit No. ESAVI-2010-05480/Ym-23). The methods were carried out in accordance with the relevant guidelines and regulations of the Finish Museum of National History. We confirm that our study complied with the ARRIVE guidelines.

The genomic DNA was isolated from the whole blood sample of each sampled individual using the ROTI^®^Prep Blood Genomic DNA MINI kit. DNA concentration was measured using the NanoDrop One Spectrophotometer (Thermo Fisher Scientific, USA) and ranged from 1.5 to 124.6 ng/μL (n = 251). For initial sex determination testing, 85 samples of nestlings and all nine adults were analysed by three primer pairs, P2/P8, 2550F/2718R, and CHD1F/CHD1R (Table 1). The remaining nestlings’ samples were sexed using CHD1F/CHD1R. Quantitative PCR was carried out in 20 μl volume containing 4 µl of genomic DNA, 2 × EliZyme™ Green MIX AddROX (Elisabeth Pharmacon, Czechia) and 10 μM of each primer. Quantitative PCR analyses for all primers were performed using QuantStudio™ Design & Analysis Software 1.5.2, following the cycling profile: 94 °C for 4 min for initial incubation, followed by 40 cycles at 94 °C for 30 s, 57 °C for 45 s, and 72 °C for 1 min^37^. Melt curve analysis followed at 60 °C for 90 s and 95 °C for 30 s^31^. A 3% agarose gel electrophoresis was performed at 100 V for 30 min to visualise the products. Moreover, applying the two methods, melt curve analysis and electrophoresis, allowed for mutual confirmation of the correctness of the individual sex determination.

**Table 1:** The names and sequences of the individual primers utilised within this study.

Primer name	Primer sequence (5′–3′)	References
P2	TCTGCATCGCTAAATCCTTT	^27^
P8	CTCCCAAGGATGAGRAAYTG	
2550F	GTTACTGATTCGTCTACGAGA	^28^
2718R	ATTGAAATGATCCAGTGCTTG	
CHD1F	TATCGTCAGTTTCCTTTTCAGGT	^29^
CHD1R	CCTTTTATTGATCCATCAAGCCT	

Six samples (three males and three females) were sequenced using all three primer pairs. If only one band was present in the gel, the amplicon was directly purified by Monarch^®^ PCR & DNA Cleanup Kit. In the case of female samples with two bands in the gel, each DNA fragment was initially extracted from the gel, followed by DNA purification using the Monarch^®^ DNA Gel Extraction Kit. SEQme Ltd., Czechia, sequenced the purified products. The obtained sequences were identified by the Basic Local Alignment Search Tool (hereafter BLAST) in the NCBI.

## Results

**Figure 1:**
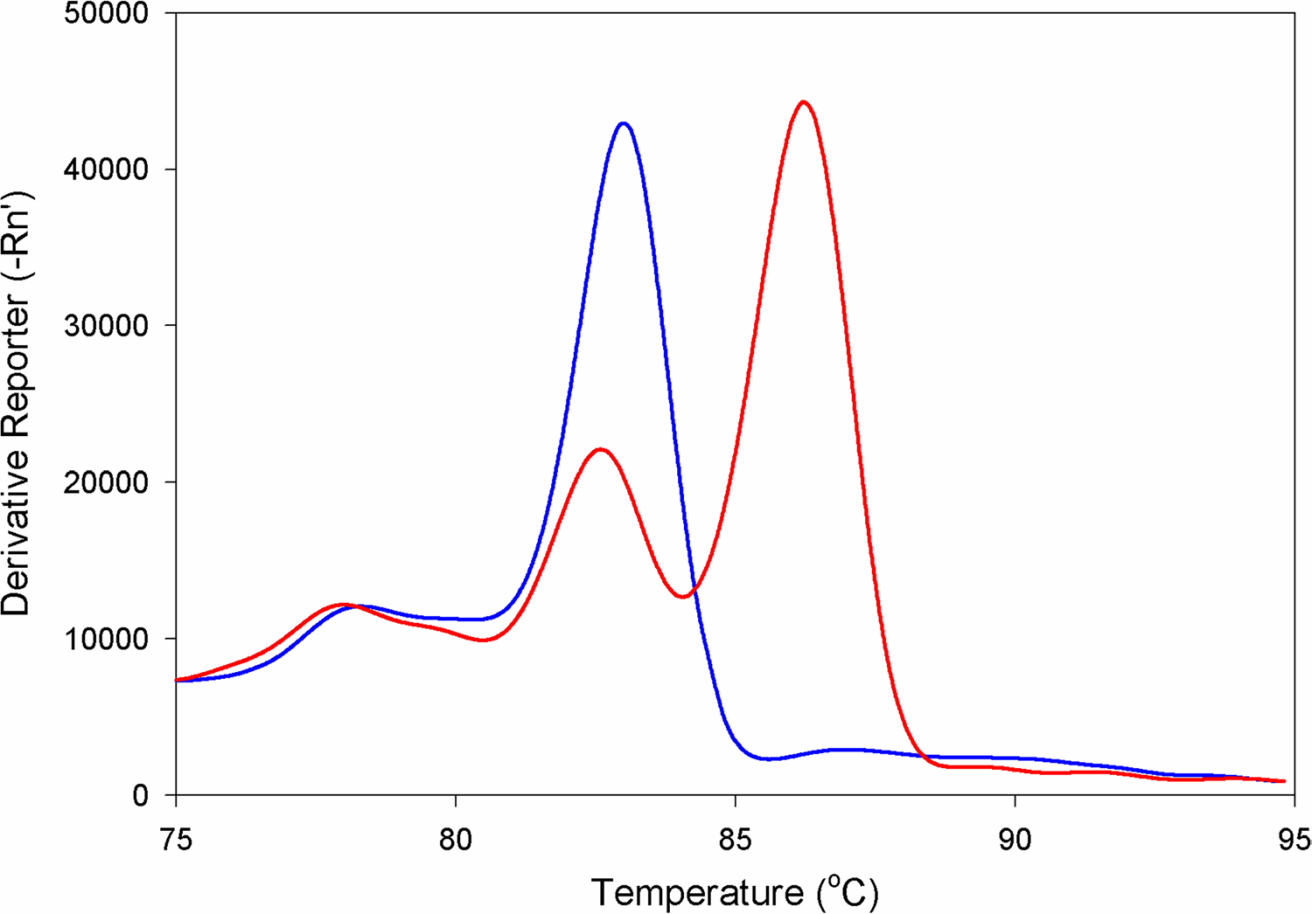
Melt curve analysis of quantitative polymerase chain reaction products from male and female Eurasian pygmy owls using primer set CHD1F/CHD1R. One peak shows the male (blue curve), and two show the female sex (red curve).

**Figure 2:**
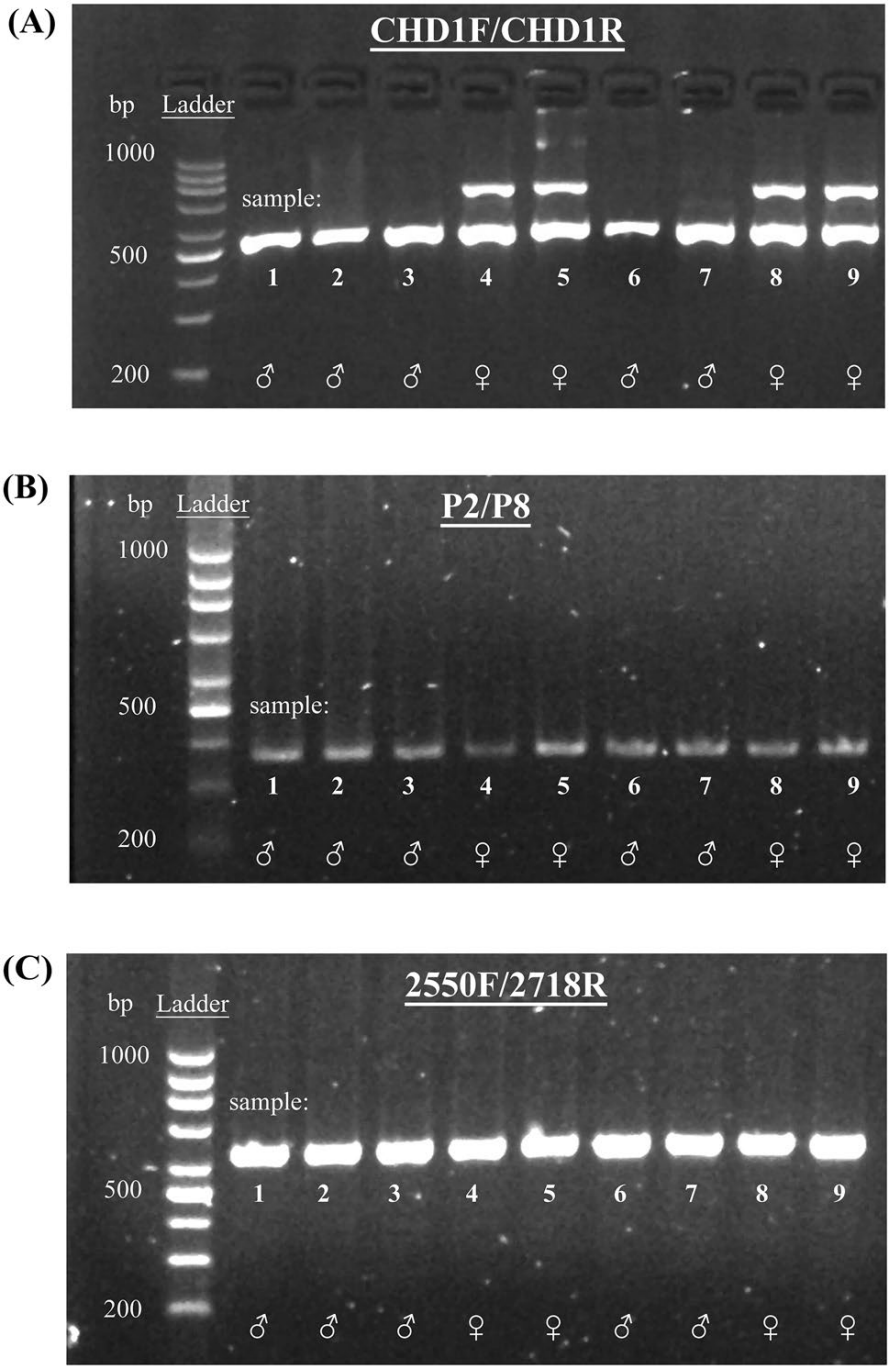
Agarose gel electrophoresis of quantitative polymerase chain reaction products of Eurasian pygmy owls using primer sets CHD1F/CHD1R (**A**), P2/P8 (**B**), and 2550F/2718R (**C**). The CHD1F/CHD1R-amplified examples demonstrate the presence of one band for males and two bands for females. The P2/P8-amplified and 2550F/2718R-amplified examples illustrate the presence of one band in both males and females, which was identical for all sampled individuals. Cropped gels are displayed. Original gels are presented in the Supplementary Information File (Supplementary Figs. 1 and 3).

Melt curve analysis of qPCR products of CHD1F/CHD1R primers depicted one peak at temperature 83 °C for males and two peaks at temperatures 83 °C and 86 °C for females (Fig. 1). Likewise, qPCR products of the same primer set displayed on 3% agarose gel electrophoresis both the one band for males (CHD1Z fragment long ∼ 500 bp—base pair) and two bands for females (CHD1Z fragment and CHD1W fragment long ∼ 900 bp) (Fig. 2A; Supplementary Fig. 1). In the case of the P2/P8, melt curve analysis of qPCR products depicted one peak at the temperature 81.5 °C for all specimens only (Supplementary Fig. 2). Furthermore, a single band was displayed on the 3% agarose gel electrophoresis only, showing a fragment of ∼ 350 bp for all specimens—both males and females (Fig. 2B; Supplementary Fig. 3). Primer set 2550F/2718R yielded similar results as the previous primer, except that melt curve analysis depicted one peak at the temperature of 83 °C for both males and females (Supplementary Fig. 4) and a single band on the electrophoresis showed a fragment of ∼ 630 bp (Fig. 2C; Supplementary Fig. 3). In total, 242 blood samples of nestlings were sexed by CHD1F/CHD1R: 131 were determined to be males and 101 to be females. The sex identification of ten juveniles failed due to poor-quality blood samples.

Furthermore, CHD1Z and CHD1W genes were successfully sequenced from male and female samples using CHD1F/CHD1R primers. Sequence from the CHD1Z gene was the only amplified fragment using 2550F/2718R primers in both male and female samples. Using the P2/P8 primers, the amplicons of CHD1Z genes from the male specimen were successfully sequenced, whereas the sequencing of amplicons from the female was unsuccessful (low-quality sequences). The sequences of CHD1Z and CHD1W from male and female samples using CHD1F/CHD1R primers were deposited into the NCBI GenBank database under accession numbers PP391271 and PP391272, respectively. Using BLAST in the NCBI database, all sequences were identified as the CHD1 genes of several owl species belonging to the same family of POs, Strigidae, with different similarity percentages (Table 2). The results from all three methods, the melt curve analysis, electrophoresis, and sequencing, were identical, confirming their mutual correctness.

**Table 2:** Identification of different quantitative polymerase chain reaction (qPCR) products after Sanger sequencing by the Basic Local Alignment Search Tool (BLAST) using the National Centre for Biotechnology Information (NCBI) database. The table presents the lengths of the DNA fragments CHD1Z and CHD1W on the chromo-helicase DNA-binding protein 1 (CHD1) gene from Z and W chromosomes produced by three sets of primers for males (M) and females (F). The fragment lengths were obtained from sequencing after quality filtering and are stated in base pairs (bp). Further, the owl species and the accession number of the identified sequences with the highest similarity percentages (per cent identity) of the CHD1 gene using the BLAST in the NCBI database are represented. All sequences identified as CHD gene from different owl species belong to the family of Strigidae.

Primer used	Sex	Fragment	Length of the fragment	Species	Per cent identity	Accession number
P2/P8	M	CHD1Z	343	*Aegolius acadicus*	96.2	DQ985262.1
	F^a^	–	–	–	–	–
2550F/2718R	M	CHD1Z	628	*Strix nebulosa*	94.4	KF601354.1
	F	CHD1Z	631	*Athene cunicularia*	93.6	DQ985254.1
CHD1F/CHD1R	M	CHD1Z	501	*Strix nebulosa*	93.4	KF601354.1
	F	CHD1Z	509	*Strix nebulosa*	93.3	KF601354.1
		CHD1W	907	*Megascops asio*	92.3	HQ593874.1

^a^The quality of sequences from female using P2/P8 primers was low. Therefore, it was impossible to determine whether the fragment presented CHD1Z or CHD1W. Both CHD1Z and CHD1W of very similar length were possibly present in the female’s qPCR product of P2/P8 primers and thus could not be distinguished. For the same reason, it was not possible to identify the sequence in the NCBI database using BLAST.

## Discussion

Our molecular analyses showed that out of the three primer sets employed, only the CHD1F/CHD1R primers provided reliable results. The primers CHD1F/CHD1R depicted one peak for males and two for females using melt curve analysis and showed one band for males and two bands for females on agarose gel electrophoresis. Samples of adults of known sex confirmed the accuracy of the primers. The other two tested primer sets, the P2/P8 and 2550F/2718R, displayed only one peak/band in all specimens, regardless of the tested individual’s sex.

Although the identification of PO sex using primers P2/P8 and 2550F/2718R remained unachievable, many other studies commonly used these primers and even described them as offering the broadest possible coverage for various Neognathae species^35,37,38,42^. Our sequencing results showed that only CHD1Z was amplified employing 2550F/2718R in both PO males and females. We speculate that the unsuccessful amplification of W-fragment in females could be caused by polymorphism on the W chromosome. A similar problem was documented in other avian species; nevertheless, polymorphism always occurred on the Z chromosome and arose only with the P2/P8-amplified products^43^. For that reason, using primer pair 2550F/2718R instead of P2/P8 was even recommended because primer pair 2550F/2718R flanks a distinct intron, which is most probably responsible for a polymorphism of fragments from the Z chromosome^43^. Nonetheless, neither of these primer pairs (P2/P8 and 2550F/2718R) produced satisfactory results in the case of POs’ sex determination.

Another potential reason for primer inaccuracy could be the slight length difference between separate Z- and W-fragments, making them undistinguishable on the agarose gel electrophoresis. This possibility appears probable with the P2/P8 primer pair in PO. We suggest that P2/P8 actually amplified both CHD1Z and CHD1W; however, due to their small length distinction, only one band appears on the agarose gel. This explanation is also supported by the low quality of the female’s sequence, which could indicate the occurrence of two unspecified fragments. A difference of less than 10 bp between fragments CHD1Z and CHD1W was exhibited, for instance, in brown wood owl (*Strix leptogrommica caligata*) and collared scops owl (*Otus lettia*) when employing primers P2/P8^29^. The sex could not be further determined in Eurasian eagle owls due to the lack of length polymorphism with the P2/P8 and 2550F/2718R primer pairs. A single band of equal size was depicted in all individuals^42,44^. Similar results to those described above were also found in other birds of prey, specifically in Accipitriformes and Falconiformes^45^. It is possible to use polyacrylamide gel or capillary electrophoresis instead of agarose gel electrophoresis because these techniques provide better separation resolution between fragments of close size^29,37,43^. However, those last methods can determine only the length of fragments and cannot render the exact sequences of DNA fragments.

In the PO, the sequence of Z-fragment measured ∼ 500 bp, while the W-fragment was 907 bp long when the primer set CHD1F/CHD1R was used (Table 2). The size disparity between the shorter CHD1Z and longer CHD1W is consistent across other owl species. Z-fragment and W-fragment were detected at 519 bp and ∼ 931 bp in collared scops owls using CHD1F/CHD1R primer^29^. Likewise, the Z-fragment lengths in our analyses, amplified by 2550F/2718R and P2/P8, were similar to other owl species. CHD1Z ranged from 600 to 650 bp in Tengmalm’s owls and great grey owls (*Strix nebulosa*) employing the 2550F/2718R primers^28^. The length of CHD1W was ∼ 1.2 kb (kilobases) in these species. The Z-fragment amplified by the P2/P8 primers from three owl species [Eurasian eagle owl, Eurasian scops owl (*Otus scops*), and barn owl (*Tyto alba*)] ranged between 360 and 364 bp^37^, which is also in accordance with our results.

The findings in owls mentioned above contrast with most other bird groups, where CHD1W is generally shorter than CHD1Z, and the differences between fragments are comparatively more minor^28,37^. For example, matching the results of 2550F/2718R, the plush-crested jays (*Cyanocorax chrysops*), Passeriformes, were characterised by Z-fragment ∼ 650 bp and W-fragment ∼ 450 bp in length^46^ and the sex of brown eared pheasants (*Crossoptilon mantchuricum*), Galliformes, were revealed by Z-fragment ∼ 600 bp and W-fragment ∼ 450 bp long^19^. Primers CHD1F/CHD1R produced 443 bp in Z chromosome amplicon and 327 bp in W one in mallard duck (*Anas platyrhynchos*), Anseriformes^29^. However, researchers must exercise caution, considering that the efficacy of the described methods varies among species. Consequently, pretesting the provided primers and the procedures for each specific species is imperative.

To conclude, we offer the protocol for molecular sex determination in PO based on melt curve analysis with amplification of the CHD1 gene. However, this sexing assay cannot be applied to POs with the primer sets P2/P8 and 2550F/2718R commonly used for bird sex determination, as they display only one DNA fragment in both males and females. In contrast, primer pair CHD1F/CHD1R provided reliable differentiation between males and females of PO, as illustrated, in addition to melt curve analysis, by electrophoretic separation of amplified fragments in agarose gel and sequencing results.

### Supplementary Information

Supplementary Figures.

## Data Availability

The raw data supporting the conclusions of this article will be made available by the authors without undue reservation. The sequences obtained in this investigation have been deposited into the NCBI GenBank database under accession numbers PP391271 and PP391272.
