# Supplementary material for: Comparison of three primer pairs for molecular sex determination in Eurasian pygmy owls (Glaucidium passerinum)
Source: Sci Rep. 2024 Jul 16;14:16397. doi: 10.1038/s41598-024-65157-3 (PMC11252122; doi:10.1038/s41598-024-65157-3)
Supplement: Supplementary file 1 — Supplementary Figures. [file 41598_2024_65157_MOESM1_ESM.pdf]

Journal: **Scientific Reports**

Comparison of three primer pairs for molecular sex determination in Eurasian pygmy owls (*Glaucidium passerinum*)

Simona Stehlíková Sovadinová[1*], Chahrazed Mekadim[2], Erkki Korpimäki[3], Jakub Mrázek[2], Marek Kouba[1,4]

[1]Czech University of Life Sciences Prague, Faculty of Agrobiology, Food and Natural Resources, Department of Ethology and Companion Animal Science, Kamýcká 129, Praha 6 – Suchdol, 165 00, Czechia

[2]Czech Academy of Sciences, Institute of Animal Physiology and Genetics, Laboratory of Anaerobic Microbiology, Vídeňská 1083, Praha 4 – Krč, 142 20, Czechia

[3]University of Turku, Department of Biology, Section of Ecology, 20014 Turku, Finland

[4]Czech University of Life Sciences Prague, Faculty of Forestry and Wood Sciences, Department of Game Management and Wildlife Biology, Kamýcká 129, Praha 6 – Suchdol, 165 00, Czechia

*Corresponding author: s.stehlikova.s@gmail.com

ORCID – Simona Stehlíková Sovadinová: 0009-0008-9431-5156; Chahrazed Mekadim: 0000-0002-5793-3501; Erkki Korpimäki: 0000-0001-7596-1955; Jakub Mrázek: 0000-0003-1907-3333; Marek Kouba: 0000-0003-2262-5733

Figures

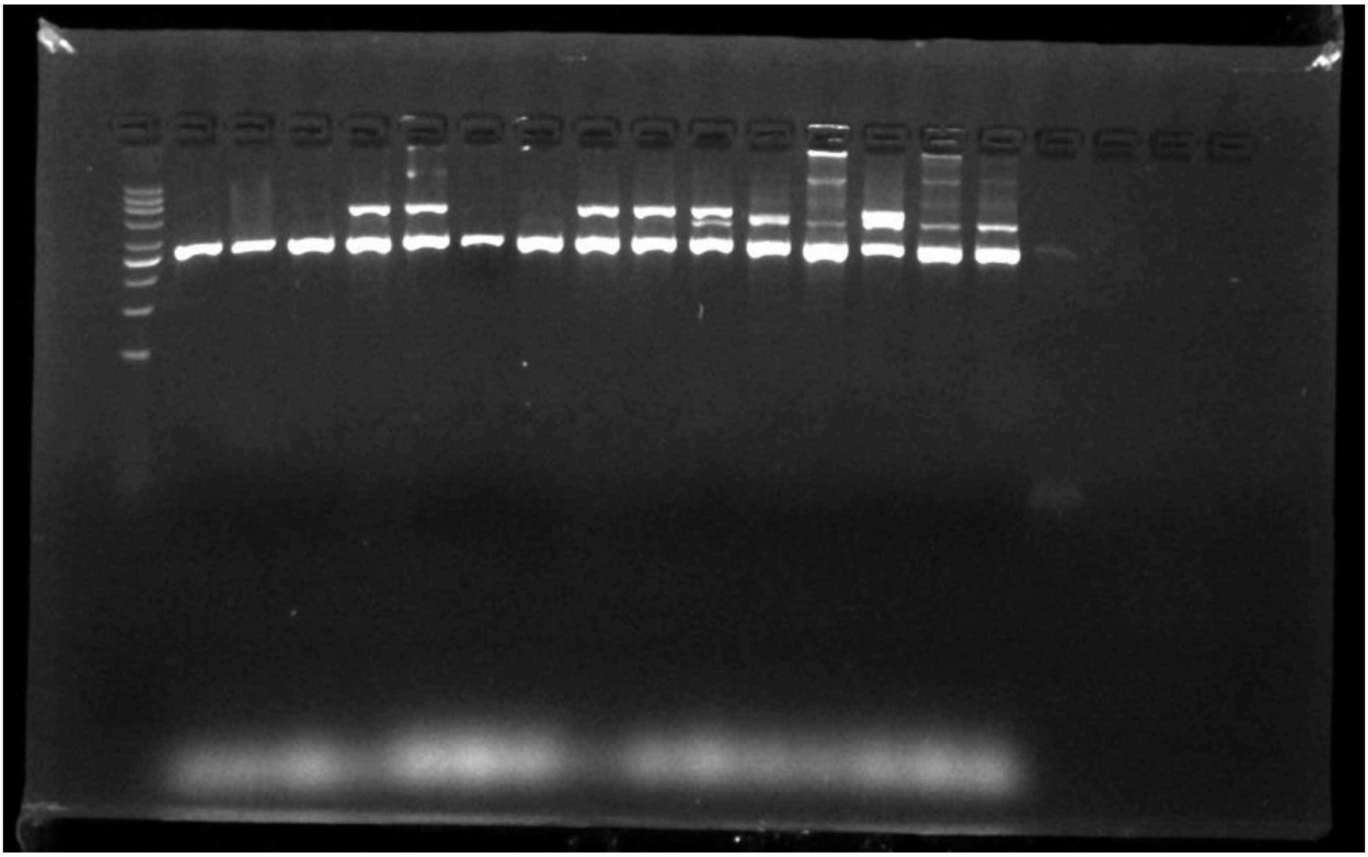

Supplementary Fig. 1. Original agarose gel from electrophoresis of PCR products of Eurasian pygmy owls using primer set CHD1F/CHD1R.

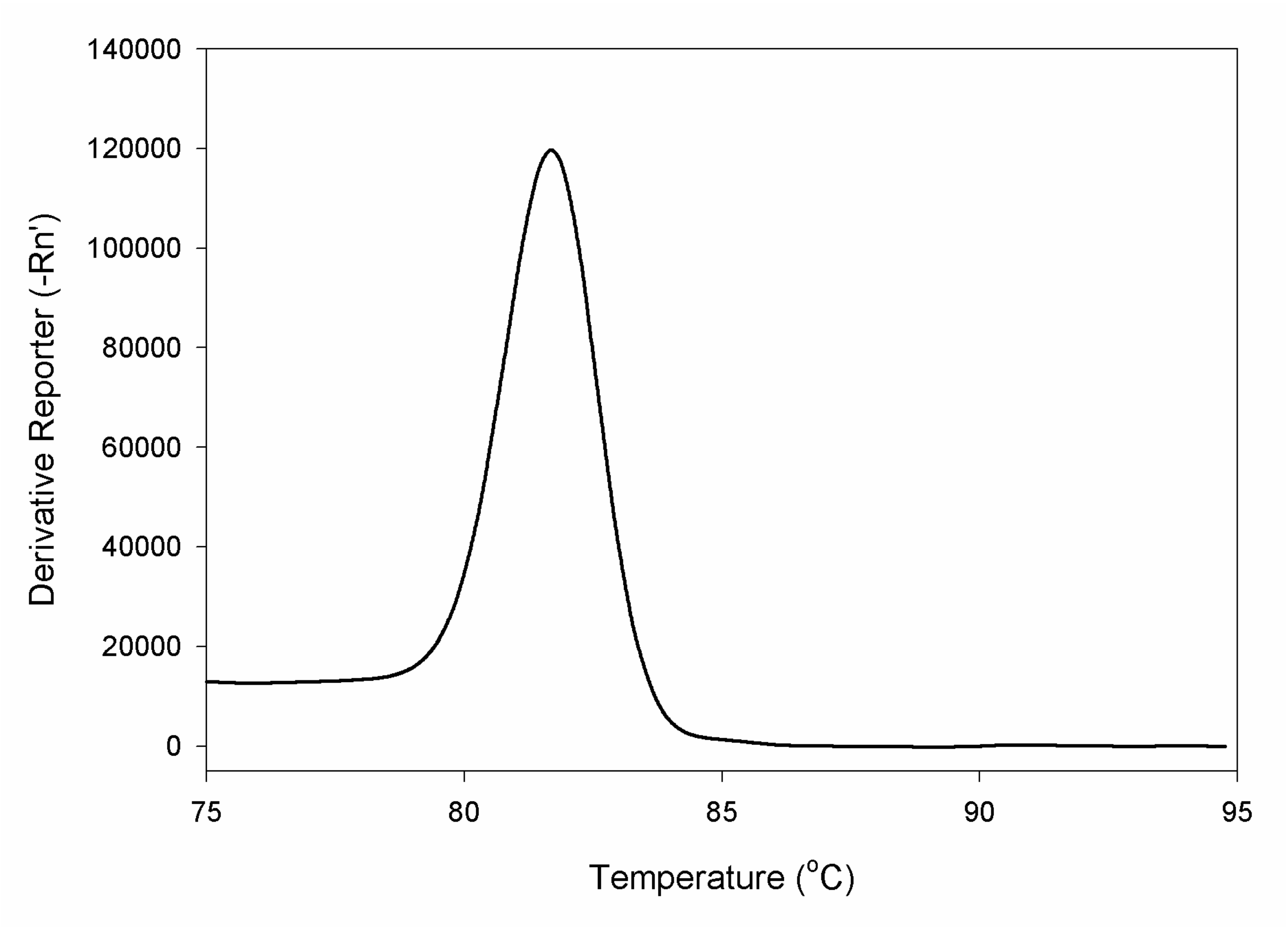

Supplementary Fig. 2. Melt curve analysis of quantitative polymerase chain reaction products from Eurasian pygmy owls using primer set P2/P8. The curve was identical for males and females.

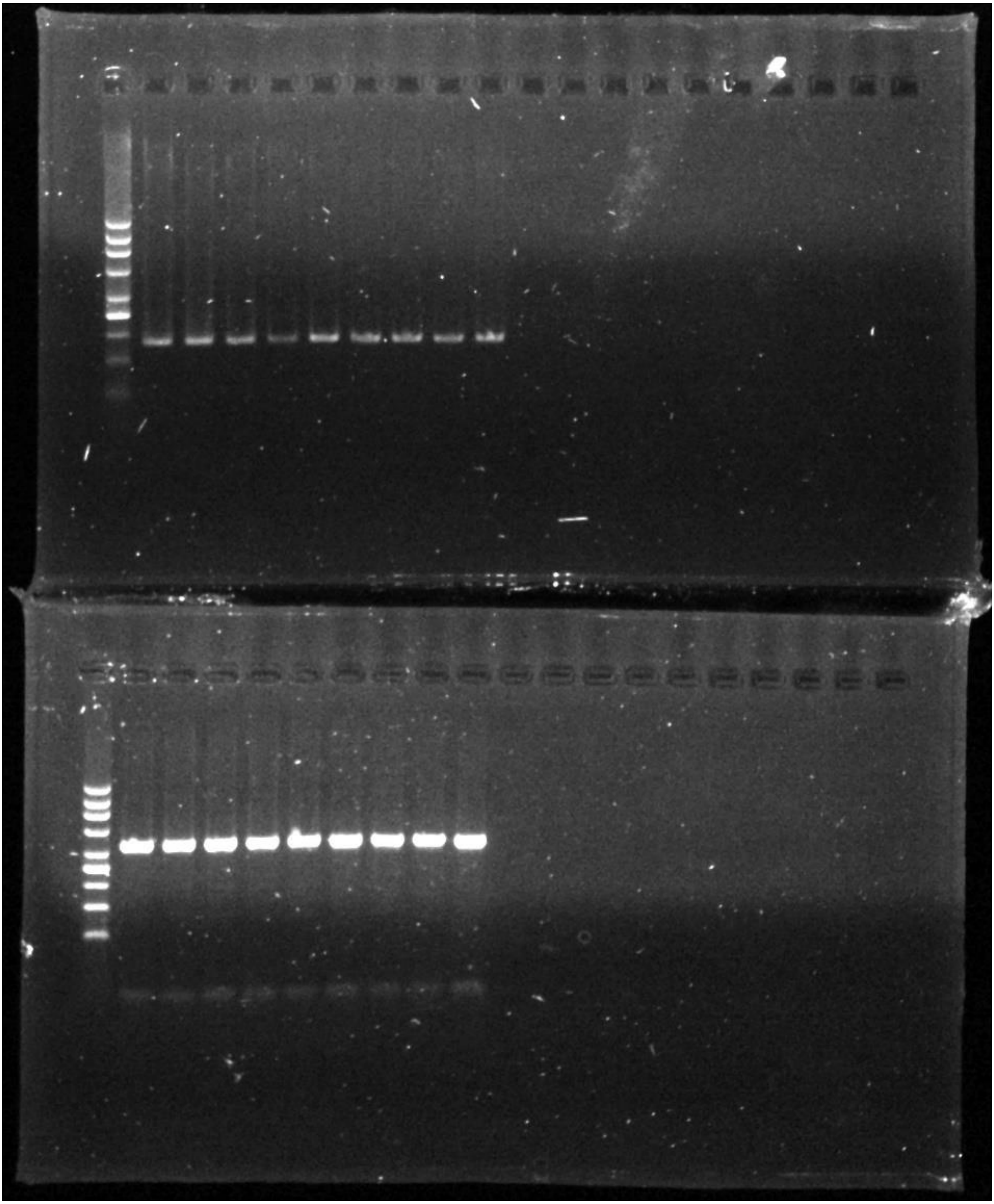

Supplementary Fig. 3. Original agarose gel from electrophoresis of PCR products of Eurasian pygmy owls using primer sets P2/P8 (upper) and 2550F/2718R (lower).

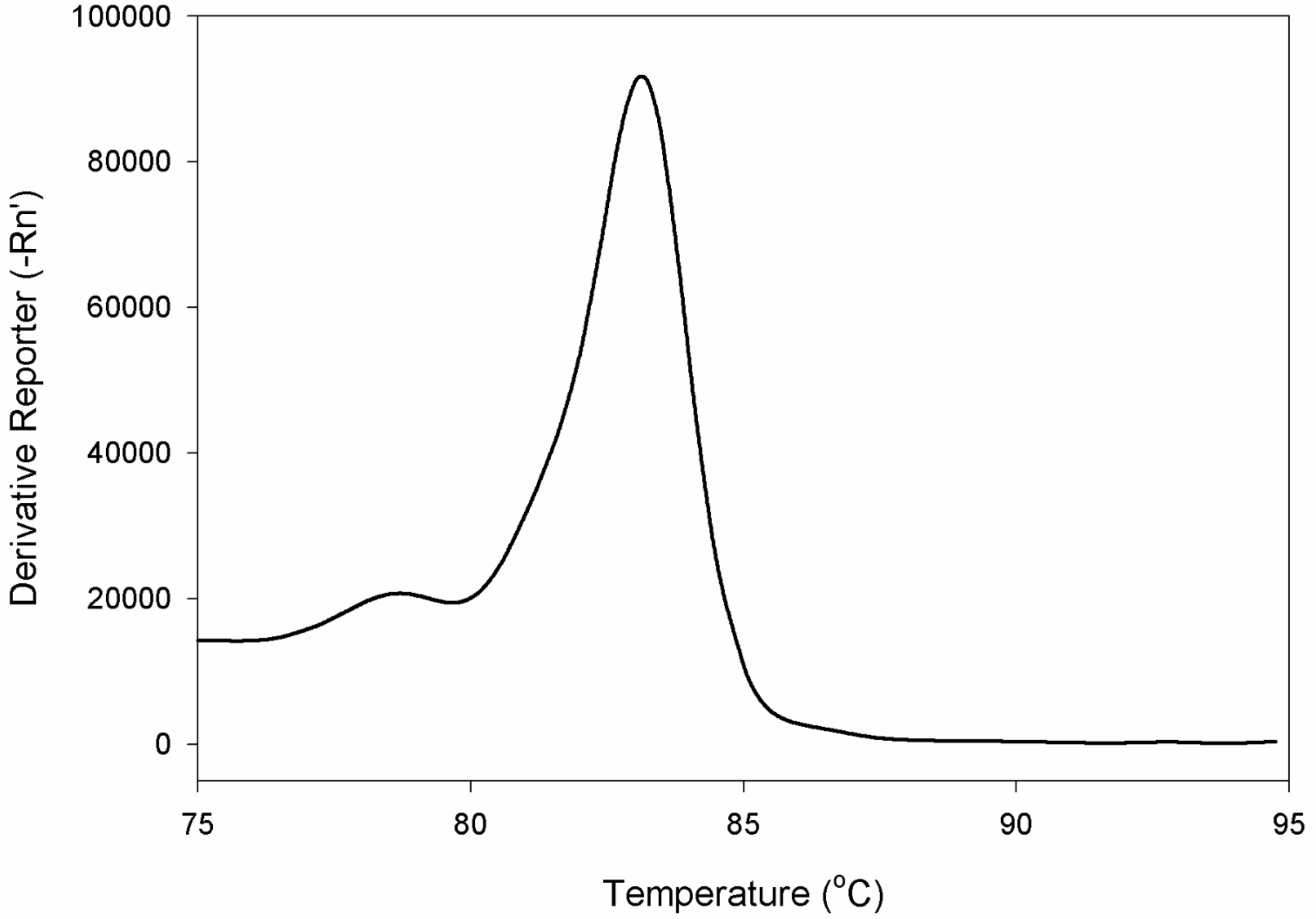

Supplementary Fig. 4. Melt curve analysis of quantitative polymerase chain reaction products from Eurasian pygmy owls using primer set 2550F/2718R. The curve was identical for males and females.
